# Survival Analysis to Estimate Association between Short-Term Mortality and Air Pollution

**DOI:** 10.1289/ehp.8311

**Published:** 2005-10-03

**Authors:** Johanna Lepeule, Virginie Rondeau, Laurent Filleul, Jean-Francois Dartigues

**Affiliations:** 1 Institut National de la Santé et de la Recherche Médicale (National Institute of Health and Medical Research), E0338 Biostatistic,; 2 Institut Fédératif de Recherche en Santé Publique (Research Federation on Public Health),; 3 Institut de Veille Sanitaire (Inter-Regional Epidemiology Cluster (Cire) of the French National Institute for Public Health Surveillance), and; 4 Institut National de la Santé et de la Recherche Médicale (National Institute of Health and Medical Research), Bordeaux, France

**Keywords:** air pollution, Cox proportional hazards model, distributed lag, mortality, short-term effect

## Abstract

**Background:**

Ecologic studies are commonly used to report associations between short-term air pollution and mortality. In such studies, the unit of observation is the day rather than the individual. Moreover, individual data on the subjects are rarely available, which limits the assessment of individual risk factors. These associations can also be investigated using case–crossover studies. However, by definition, individual risk factors are not studied, and such studies analyze only dead subjects, which limits the statistical power.

**Objective:**

We suggest that the survival analysis is more suitable when cohorts are examined with a time-dependent ecologic exposure. To our knowledge, to date this type of analysis has never been proposed.

**Design, participants, measurements:**

In the present study we used a Cox proportional hazards model to investigate the distribution over time of the short-term effect of black smoke and sulfur dioxide in 439 nonaccidental and 158 cardiorespiratory deaths among the 1,469 subjects of the Personnes Agées QUID (PAQUID) cohort in Bordeaux, France. The model has a delayed entry and a polynomial distributed lag from 0 to 5 days. Results are adjusted for individual risk factors, temperature, relative humidity, weekday, season, influenza epidemics, and a time function to control temporal trends.

**Results:**

We identified a positive and significant association between cardiorespiratory mortality and black smoke, with a 24% increase in deaths 3 days after a 10-μg/m^3^ increase in black smoke (95% confidence interval, 4–47%).

**Conclusions:**

We conclude that the Cox proportional hazards model with time-dependent covariates is very suitable to investigate simultaneously the short-term effect of air pollution on health and the effect of individual risk factors on a cohort study.

Several epidemiologic study designs are used to investigate air pollution and health, applying different methods for estimating health risks associated with variations in exposure across spatial and temporal gradients. Studies are often classified according to the type of data: individual- or aggregate-level data on exposure, health, and confounding factors. Among study designs assessing the association of short-term variations in pollution and health outcomes, the most widely used are time-series studies and case–crossover studies. Various studies have shown that concentrations of ambient air particles are associated with an increase, the same day and the day after, in all-cause mortality (Bremner et al. 1999; Peters et al. 2000; Rossi et al. 1999), respiratory mortality (Bremner et al. 1999; Rossi et al. 1999), and cardiovascular mortality (Braga et al. 2000; Bremner et al. 1999). Regression models, such as the generalized additive models with nonparametric splines or the generalized linear models with parametric regression splines, are commonly used in time-series analysis to estimate the increase in risk for a health outcome such as mortality, associated with a unit increase in ambient air pollution levels on a short-term basis (Filleul et al. 2004a). Such models make it possible to include smooth functions of time and temperature to adjust for seasonal variations, long-term trends, and temporal changes in factors that might bias the estimation of the health risk. Studies using this approach are called ecologic studies because data are aggregated and the daily number of deaths is investigated. The unit of observation is the day rather than the individual. Moreover, individual data on the subjects are rarely available, which limits the assessment of individual risk factors. In the case–crossover design, each subject is his or her own control, and air pollution levels on the dates of death (case period) are compared with those 1 week before or after death (control period) (Bateson and Schwartz 2001; Navidi and Weinhandl 2002). Consequently, all individual risk factors are inherently controlled, and their effect cannot be assessed. Individual risk factors must be taken into account to investigate air pollution and health because they can explain variations in the susceptibility or resistance among individuals to variations in air pollution concentrations. Moreover, in the case–crossover design, only subjects who have died are included in the analysis, thereby involving a loss of power whenever a cohort is available (because information about live subjects is not included in the analysis). Given that the effects on mortality associated with short-term increases in particulate air pollution are relatively slight, this loss of information cannot be neglected.

The purpose of our new approach is to treat simultaneously daily exposure to air pollution and individual risk factors, without aggregating over subjects or time. We used the Cox proportional hazards model (Cox 1972) to analyze the effect of air pollution on the short-term mortality, which has never been proposed to date. This new analysis combined the advantages of the cohort and time-series methods. The key advantage of the cohort approach is its ability to assess and to adjust for individual risk factors of susceptibility such as smoking habits, sex, and occupation, which have previously been used only to study long-term associations between air pollution exposure and health outcomes (Dockery et al. 1993; Pope et al. 1995). The key advantage of the time-series approach is to adjust for seasonal variations, long-term trends, and temporal changes for factors such as temperature, humidity, and day of the week. In this way, insights may be gained into the exposure–response relationship by allowing for simultaneous examination of the impact of both subject-specific and time-related factors on mortality. Furthermore, the power of the survival analysis is increased compared with the case–crossover approach, simply because all the subjects are studied. We used cohort data with the Cox proportional hazards model, in which exposure to air pollution is considered as a time-dependent covariate. We analyzed the distribution over time of the short-term effect of air pollution concentrations on mortality. These data were previously analyzed using the case–crossover method with a semisymmetric bidirectional design (Filleul et al. 2004b); this analysis was not completely satisfactory simply because only deaths could be studied, so a large part of the cohort was not analyzed. Moreover, with the case–crossover design, we cannot identify and assess the effects of the individual risk factors because there are inherently controlled.

A survival method taking into account all the information about the cohort seems more appropriate. In this approach, survival times are not aggregated and the Cox proportional hazards model takes into account individual factors and quantifies their effects on the association between mortality and air pollution concentration, which is impossible with the case–crossover design.

## Materials and Methods

### Study subjects.

All the subjects of the Personnes Agées QUID (PAQUID) cohort living in the urban area of Bordeaux in southwestern France were included. Data on air pollution were available only for this area. This cohort was designed to prospectively study cerebral and functional factors of aging in a representative sample of 3,777 people. Subjects were ≥ 65 years of age at inclusion and living at home in the administrative areas of Gironde and Dordogne. They were randomly selected from the general electoral lists of the administrative areas after stratification by age, sex, and urban unit. An informed consent was obtained from each participant before the study embarked. Trained psychologists interviewed the subjects at home at inclusion, in 1988. Interviews made it possible to fill out a detailed questionnaire on sociodemographic characteristics and health status. The general methodology of PAQUID has been previously published (Dartigues et al. 1992). The studied sample consisted of 1,469 subjects, of whom 543 died between 1988 and 1997.

### Health data.

Mortality data were provided by the French National Institute of Health and Medical Research, which carries out the coding of the medical causes of death according to the *International Classification of Diseases, 9th Revision* (ICD-9) (World Health Organization 1978). Causes of death corresponded to the principal cause recorded in the death register. Most studies investigate mortality from all nonaccidental causes or from broad categories of illness such as cardiovascular and respiratory diseases. To ensure a sufficient sample size, we considered only two causes of deaths: cardiorespiratory causes (ICD-9 codes 460–519 and codes 390–459) and all-cause deaths except those from accidental causes (ICD-9 codes < 800). The residence area at the time of death was recorded. Influenza data were provided by the teleprocessing national network of monitoring and information on transmissible diseases (Valleron and Garnerin 1992).

### Environmental data.

The main source of air pollution in the urban area of Bordeaux from 1988 to 1997 was motor vehicle emissions. We obtained air pollution data from the Association de Prévention de la Pollution Atmosphérique, which operated a local monitoring network from 1981 to 1997. Stations were selected to represent background inner-city air quality levels (i.e., stations not directly influenced by industrial or road traffic sources of pollution). The ambient urban stations measures had to be sufficiently correlated (i.e., correlation > 0.70) and to have sufficiently similar mean levels of pollution. Four stations corresponded to these criteria for black smoke (BS), measured by reflectometry, and six for sulfur dioxide-strong acidity (SO_2_-AF) measured by the acidimetric method. We constructed exposure indicators by calculating the arithmetic mean of daily concentrations recorded in the selected ambient urban stations. Meteorologic data (daily temperature and daily relative humidity) were provided by Météo-France, Bordeaux Merignac, France.

### Analytical approach.

The Cox proportional hazards model is widely used for statistical analysis in epidemiology studies, particularly owing to its simple calculation and clear interpretation. It provides a parametric relation between the risk factors included in the model and the survival distribution without imposing a parametric form. In failure-time analysis, continuous time-dependent covariates are rarely used. In this study, the measurements of air pollutant concentrations were time-dependent covariates with 3,653 different values over the 10 years of follow-up. For each cause of death and each pollutant, we fitted a time-dependent Cox proportional hazards model, modeling the relative risk of death for a 10-μg/m^3^ increase in pollutant concentration. Age was chosen as the basic time scale for two major reasons. First, it allows the study of age, an important risk factor of death, without making parametric assumptions about the effect of this variable. Second, the effect of air pollution is not identifiable when calendar time is used. Using age instead of calendar time solves the problem. We then used a time-dependent Cox proportional hazards model with delayed entry (Klein and Moeschberger 1997) represented by the risk function at age *a* for a subject *i*:


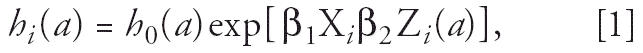


where *a* was the age, *h*_0_(*a*) was the unspecified baseline hazard function, X*_i_* was the vector of time-independent explicative variables (sex, occupational exposure, and cigarette smoke exposure), and *Z_i_*(*a*) was the vector of time-dependent explicative variables (air pollution, temperature, humidity, influenza epidemics, season, and day of the week) β*_j_*, *j* = 1,2 being the vector of the unknown model parameters. The risk set was calculated for each age of death with *a*_1_, *a*_2_, . . . *a*_De_, corresponding to the ordered ages of death observed in the sample. Because we used a Cox proportional hazards model, a first condition to be included in the risk set at age *a_i_* was to be alive until this age. The second condition to be included in the risk set at age *a_i_* was to be younger than *a_i_* at inclusion. This ensured that we knew air pollution exposure of all the subjects included in the risk set at age *a_i_*.

Because the death on a given day not only is a function of the same-day exposures to a pollutant but also is affected by exposures during a certain lag period (a few days), we used a distributed lag model. Distributed lag models have been used for decades in social sciences (Judge et al. 1980), and several researchers (Pope et al. 1992; Pope and Schwartz 1996; Rondeau et al. 2005; Schwartz 2000; Zanobetti et al. 2000) have recently described the use of this approach in epidemiology for generalized additive models or for generalized linear models. We have adapted the distributed lag structure on the survival models:


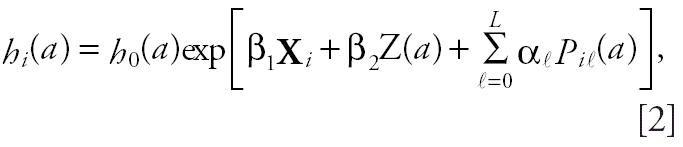


where air pollution concentrations were represented by *P_ia_*, and α_ℓ_ indicated the magnitude of the effect of air pollution at day ℓ with ℓ = 1, . . . , *L*, and *L* being the number of lag days for the pollutant covariates. We chose the length of the lag to minimize Akaike’s Information Criterion (AIC) (Akaike 1973) for each of the four models. AIC represents improvement in model fit versus the number of degrees of freedom that produced that improvement.

Given that exposure is serially correlated on several subsequent days, the above unconstrained distributed lag model will have a high degree of multicollinearity (Schwartz 2000). The collinearity among the lagged coefficients will lead to unstable estimation of the α_ℓ_ values with an increase in the variance of each estimator. Following the work of Almon (1965), we assumed that the effects α_ℓ_ of the lagged exposure in the previous model (2) followed a polynomial of sufficient degree *D*, that is,


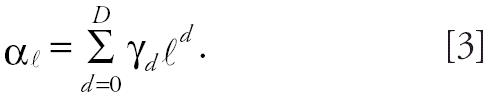


We used a constrained model. This implied that the effects of air pollution were distributed over the previous *L* days following a polynomial function of degree *D*. The quantity α_ℓ_ was then interpretable as the polynomially smoothed estimate of the effect of air pollution on lagged ℓ days, and their sum, 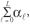, was the overall effect of air pollution over the entire lag period. Although we know that the effect of air pollution concentrations is distributed over time, we made no assumption about the form of the effect on days close together, which allows for a wide variety of lag structures. The polynomial distributed lag model allows us to adjust for nonlinear effects that are more in line with reality. This approach has the advantage of reducing both the collinearity and the number of parameters to be estimated via the assumed polynomial structure. In our case, the number of parameters was small, but when the lag is longer this advantage cannot be neglected. The explanatory power of air pollution on daily deaths being modest, parsimony in the degree of the polynomial was necessary. Polynomial degree can be chosen arbitrarily or by AIC but should not exceed 3 in most cases (Schwartz 2000; Zanobetti et al. 2000). In fact, a second or third degree offers sufficient flexibility in most cases of distributed constrained lag models (Pope and Schwartz 1996). For these reasons and so as not to multiply the number of tests, we chose a second-degree polynomial.

### Individual risk factors and potential modifiers.

We obtained results after adjusting for individual risk factors (sex, cigarette smoke exposure, and occupational exposure) and for time-dependent confounders (temperature, humidity, influenza epidemics, season, and day of the week).

An ascending method was applied to include individual risk factors in each of the four models according to the level of significance (< 25%). Individual factors were considered constant on average during the 10-year study period because subjects were ≥ 65 years of age so they did not work anymore and their occupational exposure could not change. We defined three classes for occupational exposure: never worked, white collar, and blue collar. This factor represents direct effect of occupational exposure on mortality, but also indirect effects such as lifestyle and life habits strongly depend on the occupational category.

Status about cigarette smoke exposure was defined at the inclusion in the study such as nonsmoker, ex-smoker, or current smoker. This factor was considered constant during the 10 years of follow-up because there are few changes in smoking habits among individuals ≥ 65 years of age. Generally, changes occurring after 65 years of age concern subjects who stop smoking but maintain a risk due to their former smoking exposure rather than subjects who start smoking. This assumption of constant individual factors could lead to possible bias, but with a limited impact because we assumed here a long-term effect of smoking. If the study period had been longer or the subjects younger, it would have been necessary to take into account the evolution of these factors over time.

We also analyzed the mobility of the subjects. Subjects were censored at the exact date of moving house. When we did not have precise information on the moving, they were censored at their last follow-up before moving. Mortality and pollution indicators undergo temporal variations due to factors known or unknown.

Such changes may appear in the long term (annual variations) or medium term (seasonal variations, weekly). We applied an ascending method to include potential time-dependent confounders in each of the four models according to the level of variation of the other estimated parameters (25% at least) already included in the model. Minimal temperature and relative humidity were included using the average on the selected lag for each model. Seasons were represented by a binary variable: spring–summer (21 March–20 September) versus fall–winter (21 September–20 March). Influenza epidemics were defined on the basis of their graphical description when more than 300 cases occurred in a week and were treated as a dichotomous variable. In addition, we introduced in each model an unspecified function of time to take into account the long-term time trends in the data. It was estimated by a truncated power basis spline (Heuer 1997), defined by *m* knots. We used five equidistant inner knots during the 10-year study period. We also tested other combinations of number and position of knots to assess the sensitivity of the results.

Analyses were conducted with the SAS software (version 8.2; SAS Institute Inc., Cary, NC, USA).

## Results

During the study period, the mean level of BS was 17.0 μg/m^3^ (SD = 10.6 μg/m^3^), with a minimum of 1.8 μg/m^3^ and a maximum of 99.0 μg/m^3^. For SO_2_-AF, the mean level was 10.3 μg/m^3^ (SD = 6.6 μg/m^3^), varying between 0.0 and 64.6 μg/m^3^. The correlation between daily levels of BS and SO_2_-AF was 0.67. During the same period, mean for minimal temperature was 9.3°C, and mean relative humidity was 59.9%. Among the 543 deceased subjects (248 women and 295 men), we studied 439 deaths from nonaccidental causes and 158 from cardiorespiratory causes (127 cardiac and 31 respiratory). For all nonaccidental causes, 50% of deceased subjects were > 83 years of age. Table 1 describes the main characteristics of the 1,469 subjects included in the analysis.

By univariate analysis, the data did not demonstrate a significant association between mortality and educational level. Mortality differed between occupational exposure categories. After adjustment for sex, smoke exposure, or air pollution concentration, the estimated risk remained higher but not statistically significant for blue-collar workers. Because its confounding role is recognized in the literature, we forced occupational exposure into the models.

**Figure 1:**
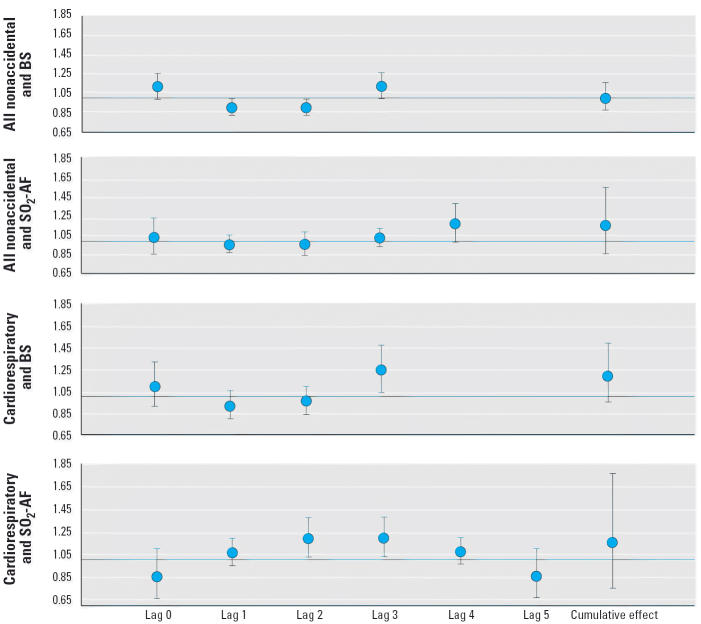
Effect and 95% CIs of a 10-μg/m^3^ increase in air pollution on death using a Cox proportional hazards model with a second-degree polynomial–distributed lag model, adjusted for meteorologic variables.

Whatever the causes of mortality or the pollutant studied, the Cox proportional hazards model with the polynomial distributed lag period selected by the AIC did not demonstrate any significant cumulative effect after adjustment for individual factors and temporal confounders. Figure 1 shows the estimated cumulative effect and the estimated effect of each single day’s exposure to BS and SO_2_-AF across 5 days and for different causes of death.

For all nonaccidental deaths and according to the AIC, lags of 3 and 4 days were selected to represent the association with BS and SO_2_-AF, respectively (Table 2). Table 2 summarizes the results of all nonaccidental death analyses adjusted for individual factors and temporal confounders. As expected, women had a lower risk of death than did men [rate ratio (RR) = 0.61; 95% confidence interval (CI), 0.46–0.79], and smokers and ex-smokers had a risk of death about 50% greater than nonsmokers. For single-day exposure, there was a greater risk of death for the third day after exposure to BS (RR = 1.12; 95% CI, 0.99–1.26) and for the fourth day after exposure to SO_2_-AF (RR = 1.17; 95% CI, 0.99–1.39), but these associations were not significant at 5%. A protective effect for the first and second days after exposure to BS was found, which is very surprising and probably due to an accentuation of the polynomial structure. The estimated effects of the first and second days’ exposure to SO_2_-AF were found to be negative.

A lag 3 for BS and a lag 5 for SO_2_-AF were selected to examine the association of these two pollutants with deaths from cardiorespiratory causes (Table 3). Concerning individual characteristics, the same estimated risk of death was observed for women versus men as with all nonaccidental deaths. The estimated effect of smoking was greater for cardiorespiratory deaths than for all nonaccidental deaths. The nonsignificant effect for current smokers probably occurred because there were only 17 subjects in this class. When adjusted for individual factors and temporal confounders, results according to single-day exposure showed that a 10-μg/m^3^ increase in BS was associated with an estimated 24% (RR = 1.24; 95% CI, 1.04–1.47) increase in cardiorespiratory mortality 3 days later. The same increase in SO_2_-AF was associated with an estimated 19% excess of deaths on the second and third days after exposure (RR = 1.19; 95% CI, 1.03–1.37).

### Sensitivity.

According to the AIC, introduction of the unspecified function of time improved the fit of the four models. To check the stability of our results, we tested both a third-degree function and an increase up to 16 inner knots for one of the four models. No appreciable differences in the estimated associations were observed. We also explored the influence of the degree of the polynomial lag structure and then replaced the second degree of the polynomial lag structure by a third degree. The estimated associations between air pollution and mortality were unchanged for all the models except for all nonaccidental deaths and BS, which had a RR < 1. For this association, first- and second-day exposures became nonsignificant with the third degree, whereas they were significantly associated with a second-degree polynomial lag structure (Table 2).

To validate the assumption that the effects of air pollution on mortality were not distributed beyond 5 days, we tested a lag period of 10 and 15 days for the association between cardiorespiratory mortality and SO_2_-AF. The models were no longer statistically satisfactory. The cumulative effects were very similar for 5, 10, and 15 days, but the CIs increased with the number of days. When we increased the lag period to 10 or 15 days, the individual effects of each single day were no more significant and the curve of the distributed lag effects tended to be smoother. Moreover, this effect reached zero after the fifth day of the lag period and became negative, as reported in Schwartz (2000) and the Institut de Veille Sanitaire (2001). We tested the proportional hazard assumption by using an interaction between age and different variables, according to the log-likelihood ratio test. This assumption was valid for all fixed variables (time independent) of all models except for cigarette smoke exposure (*p* = 0.05) in the two models concerning all nonaccidental mortality.

## Discussion

We found a positive association between short-term variations in BS and SO_2_-AF levels and mortality among persons ≥ 65 years of age. Several studies have found some effects of particulate air pollution on mortality outcomes. Schwartz (2000) observed an association between daily deaths of persons ≥ 65 years of age for all causes with particulate matter < 10 μm aerodynamic diameter (PM_10_), a result confirmed by Katsouyanni et al. (2001) in Europe with BS in the Air Pollution and Health: a European Approach (APHEA-2) study. In London, Bremner et al. (1999) showed a significant association between BS and respiratory and cardiovascular mortality. They also showed that SO_2_-AF pollution was significantly associated with respiratory mortality among the elderly.

Our findings point to a significant association between cardiorespiratory mortality and air pollution among the elderly only for the third-lag day with BS and for the second- and third-day lags with SO_2_-AF. In France, the Institut de Veille Sanitaire (2001) analyzed the time series of deaths due to cardiovascular and respiratory diseases in nine French cities with a third-degree polynomial lag structure from 0 to 5 days. This study showed that BS association with cardiovascular deaths was strongest for lag 3, but there was no significant effect with respiratory mortality. The association between SO_2_-AF and cardiovascular deaths was strongest for lags 1 and 2, and no significant association was observed with respiratory mortality. Bremner et al. (1999) explored different lag periods in the relationship between air pollution and mortality for cardiovascular and respiratory causes without polynomial structure. They found a greater risk of death associated with BS for cardiovascular causes for lag 1 and for respiratory causes for lag 3. Concerning SO_2_-AF, the first 3 days of the lag were positively associated with respiratory mortality, but no association was observed with cardiovascular deaths. Moreover, a study by Téllez-Rojo et al. (2000) also found a greater risk of death for respiratory causes outside medical units for lag 3 of PM_10_ among elderly subjects in Mexico City.

Concerning all nonaccidental deaths, our analysis found positive associations especially for lag 3 for BS and lag 4 for SO_2_-AF, which were at the limit of significance, as opposed to the negative effect of lags 1 and 2. This result cannot be explained in terms of the French health care system delaying the deaths. We also tested an unconstrained model (by adjusting directly on each lag day), and the effects were negative but not significant. We believe that the significance of this result could be due to the polynomial structure of the effect of the pollutant. Bremner et al. (1999) found nonsignificant results with all-cause mortality. However, the Institut de Veille Sanitaire (2001) study found a significant association between the first 4 days of the lag for BS and SO_2_-AF. On the contrary, we did not find any significant cumulative effects, but the risks of death of a single day’s exposure that we observed were greater than in the other studies. Filleul et al. (2004a) analyzed the time series of deaths due to respiratory, cardiovascular, and all nonaccidental causes among the elderly over the period 1988–1997 in Bordeaux City. They used generalized additive models with a cumulated lag period of 5 days and found concordant results with ours. The increase in respiratory mortality cumulated over 5 days’ lag was 9.2% (95% CI, 3.4–15.3%) for a 10-μg/m^3^ increase in BS. Our study showed an increase in cardiorespiratory mortality of 23.7% (95% CI, 3.9–47.2%) for lag 3. Concerning exposure to SO_2_-AF, Filleul et al. (2004a) showed a 20.6% (95% CI, 9.3–33.2%) excess of respiratory mortality, and we found a 19.0% (95% CI, 3.1–37.4%) excess of cardiorespiratory mortality for the second and third days after exposure.

The results obtained with the survival analysis are very similar to those obtained by the case–crossover method for exposure to BS (Filleul et al. 2004b) in the same population. Filleul et al. used a restricted distributed lag model with a polynomial effect of the pollutant. They chose a second degree for the polynomial, according to the AIC and they chose an *a priori* lag period of 0–3 days before the event based on the literature. Their data did not demonstrate any cumulative effect after adjustment for meteorologic data (daily temperature and relative humidity). Their odds ratios (ORs) for the cumulative effect and for all nonaccidental and cardiorespiratory mortality were, respectively, 0.79 (95% CI, 0.62–1.02) and 0.89 (95% CI, 0.59–1.34) for a 10-μg/m^3^ increase in BS. Nevertheless, they found an association between the third lag day and all nonaccidental mortality (OR = 1.19; 95% CI, 0.99–1.43), which was significant for cardiorespiratory mortality (OR = 1.30; 95% CI, 1.01–1.68). Therefore, the present results using survival analysis are concordant with those obtained with the case–crossover analysis for the lag period, for the degree of the polynomial, and for the level of the risks. The CIs are more restricted with the Cox proportional hazards model. Therefore, we believe that the Cox proportional hazards model should be applied when a cohort is available because survival analysis exploits all available information and increases the power of the study. Moreover, it enables identification and adjustment for individual risk factors. By using the Cox proportional hazards model where age is the time scale, it is possible to adjust non-parametrically for age. This method should prove particularly useful in the future to simultaneously analyze the short- and long-term effects of air pollution.

## Figures and Tables

**Table 1:** Characteristics of the PAQUID cohort living in the urban area of Bordeaux, 1988–1997.

	Deaths		
Characteristic	All nonaccidental causes (*n* = 439)	Cardiorespiratory causes (*n* = 158)	All subjects (*n* = 1,469)
Age at death [years, median (minimum–maximum)]	83.1 (66.1–106.1)	84.2 (67.3–102.9)	
Sex (%)			
Male	48.5	51.9	38.3
Female	51.5	48.1	61.7
Family (%)			
Living alone	49.9	46.2	47.0
Living in couple	50.1	53.8	53.0
Educational level (%)			
Without primary school diploma	28.9	27.2	25.5
Primary school diploma or secondary not validated	57.4	59.5	59.9
Secondary validated or higher	13.7	13.3	14.6
Occupational exposure (%)			
Never worked	11.8	7.0	11.1
White collar	40.8	44.9	45.2
Blue collar	47.4	48.1	43.7
Smoking habits (%)			
Nonsmoker	53.3	49.4	62.3
Ex-smoker	34.6	39.2	26.8
Current smoker	11.8	10.8	10.5

**Table 2:** Adjusted all-nonaccidental mortality RR estimates from Cox proportional hazards models with a polynomial distributed lag effect for a 10-μg/m^3^ increase in air pollution (BS and SO_2_-AF), Bordeaux, France, 1988–1997.

	BS^a^	SO_2_-AF^b^
Characteristic	RR (95% CI)	RR (95% CI)
Female vs. male	0.61* (0.46–0.79)	0.61* (0.47–0.79)
Occupational exposure vs. never worked		
White collar	0.77 (0.55–1.08)	0.77 (0.55–1.08)
Blue collar	0.97 (0.70–1.34)	0.97 (0.70–1.34)
Smoking habits vs. nonsmoker		
Ex-smoker	1.50* (1.14–1.97)	1.50* (1.14–1.97)
Current smoker	1.65* (1.17–2.32)	1.65* (1.17–2.32)
Distributed effect of air pollution		
Lag 0	1.11 (0.98–1.25)	1.03 (0.86–1.24)
Lag 1	0.90* (0.82–0.98)	0.96 (0.88–1.06)
Lag 2	0.90* (0.82–0.99)	0.96 (0.85–1.09)
Lag 3	1.12 (0.99–1.26)	1.03 (0.94–1.12)
Lag 4	—	1.17 (0.99–1.39)
Cumulative effect	1.00 (0.87–1.16)	1.16 (0.86–1.55)

a Adjusted for temperature, day of week, and function of time.

b Adjusted for temperature, humidity, day of week, and function of time.

* *p* < 0.05.

**Table 3:** Adjusted cardiorespiratory mortality RR estimates from Cox proportional hazards models with a polynomial distributed lag effect for a 10 μg/m^3^ increase in air pollution (BS and SO_2_-AF), Bordeaux, France, 1988–1997.

	BS^a^	SO_2_-AF^b^
Characteristic	RR (95% CI)	RR (95% CI)
Female vs. male	0.65 (0.42–1.01)	0.65 (0.42–1.01)
Occupational exposure vs. never worked		
White collar	1.34 (0.68–2.63)	1.34 (0.68–2.62)
Blue collar	1.55 (0.80–2.98)	1.53 (0.80–2.95)
Smoking habits vs. nonsmoker		
Ex-smoker	1.85* (1.18–2.89)	1.84* (1.18–2.88)
Current smoker	1.75 (0.97–3.16)	1.75 (0.97–3.16)
Distributed effect of air pollution		
Lag 0	1.09 (0.91–1.32)	0.84 (0.65–1.10)
Lag 1	0.92 (0.80–1.05)	1.06 (0.94–1.19)
Lag 2	0.96 (0.84–1.10)	1.19* (1.03–1.37)
Lag 3	1.24* (1.04–1.47)	1.19* (1.03–1.37)
Lag 4	—	1.07 (0.95–1.19)
Lag 5	—	0.85 (0.66–1.10)
Cumulative effect	1.19 (0.95–1.47)	1.15 (0.75–1.77)

a Adjusted for temperature, day of week, and function of time.

b Adjusted for temperature, and function of time.

* *p* < 0.05.
